# The Translocation Domain of Botulinum Neurotoxin A Moderates the Propensity of the Catalytic Domain to Interact with Membranes at Acidic pH

**DOI:** 10.1371/journal.pone.0153401

**Published:** 2016-04-12

**Authors:** Anne Araye, Amélie Goudet, Julien Barbier, Sylvain Pichard, Bruno Baron, Patrick England, Javier Pérez, Sophie Zinn-Justin, Alexandre Chenal, Daniel Gillet

**Affiliations:** 1 CEA, iBiTec-S/SIMOPRO, CEA-Saclay, Paris Saclay University, LabEx LERMIT, F-91191 Gif-sur-Yvette, France; 2 Institut Pasteur, Proteopole, Plateforme de Biophysique des Macromolécules et de leurs Interactions (PFBMI), 25–28 rue du Dr Roux, F-75724 Paris cedex 15, France; 3 Synchrotron Soleil, BP 48, F-91192 Gif-sur-Yvette Cedex, France; 4 CEA, iBiTec-S/LBSR, CEA-Saclay, F-91191 Gif-sur-Yvette, France; 5 Institut Pasteur, Unité de Biochimie des Interactions Macromoléculaires, UMR 3528, 25–28 rue du Dr Roux, F-75724 Paris cedex 15, France; Institute Pasteur, FRANCE

## Abstract

Botulinum neurotoxin A (BoNT/A) is composed of three domains: a catalytic domain (LC), a translocation domain (H_N_) and a receptor-binding domain (H_C_). Like most bacterial toxins BoNT/A is an amphitropic protein, produced in a soluble form that is able to interact, penetrate and/or cross a membrane to achieve its toxic function. During intoxication BoNT/A is internalized by the cell by receptor-mediated endocytosis. Then, LC crosses the membrane of the endocytic compartment and reaches the cytosol. This translocation is initiated by the low pH found in this compartment. It has been suggested that LC passes in an unfolded state through a transmembrane passage formed by H_N_. We report here that acidification induces no major conformational change in either secondary or tertiary structures of LC and H_N_ of BoNT/A in solution. GdnHCl-induced denaturation experiments showed that the stability of LC and H_N_ increases as pH drops, and that H_N_ further stabilizes LC. Unexpectedly we found that LC has a high propensity to interact with and permeabilize anionic lipid bilayers upon acidification without the help of H_N_. This property is downplayed when LC is linked to H_N_. H_N_ thus acts as a chaperone for LC by enhancing its stability but also as a moderator of the membrane interaction of LC.

## Introduction

Botulinum neurotoxin (BoNT) secreted by *Clostridium botulinum* is the causative agent of botulism, a neuroparalytic disease. There are seven serologically distinct BoNT isoforms designated as A to G [1]. These toxins are considered to be the most potent among bacterial, animal, plant toxins and chemical compounds. As there is no available specific cure or vaccine against botulism, BoNTs are also considered as a potential biological weapon [2]. It is also a remarkable cure for numerous neuromuscular and neuronal diseases [3]. BoNTs are synthesized as a single soluble polypeptide chain of 150 kDa. This inactive precursor is cleaved either by a clostridial or tissue protease generating two polypeptides chains linked through a disulfide bound. The light chain (LC, 50 kDa) corresponds to the catalytic domain with a zinc endopeptidase activity [3–5]. The heavy chain (HC, 100 kDa) encompasses two functional domains: the translocation domain at the N-terminus (H_N_) and the receptor-binding domain at the C-terminus (H_C_) [4,6,7]. The three domains are critical for cell intoxication by BoNTs. H_C_ allows recognition of neuronal cells by a double anchorage to a ganglioside and a protein receptor [8–11]. Following binding to its receptors, BoNT is internalized through clathrin-dynamin mediated endocytosis of recycling neurosecretion vesicles [12]. Inside the endocytic compartments, H_N_ assists the translocation of LC into the cytosol, upon acidification [13–15]. LC cleaves SNARE proteins involved in neurotransmitter release leading to the inhibition of acetylcholine release at the neuromuscular junction, resulting in flaccid paralysis [16].

The translocation, a key step of the intoxication, is still poorly understood. This step raises the issue of the physico-chemical and structural properties used by amphitropic proteins to interact and cross biological membranes. Among bacterial toxins, several mechanisms can be found such as conformational change and oligomerization for the pore-forming toxins [17]. Translocating toxins like BoNT/A have a domain, H_N_, dedicated to the membrane crossing of their catalytic domain H_N_. H_N_ may act as a channel that would provide a way through the membrane for an unfolded LC; this is the case for the anthrax toxin [18]. For the diphtheria toxin, H_N_ acts like a chaperone that helps LC to achieve a partially folded state necessary for membrane binding and penetration [19].

The details of LC membrane-crossing mechanism remain largely unknown. In the past decade based on electrophysiology measurements, it has been proposed that H_N_ could ensure a dual channel and chaperone activity. According to this model, LC would unfold through the channel formed by H_N_ to reach the cytosol [20,21]. An alternative model suggests that H_N_ could constitute a hydrophilic “cleft” that would allow the hydrophilic regions of LC to cross the membrane [15,22]. The aims of this study were to characterize the capacity of LC and H_N_ of BoNT/A to interact and penetrate a membrane and to determine how LC and H_N_ of BoNT/A influence each other during the successive steps leading to membrane binding and penetration as pH drops. We investigated by spectroscopic methods the structure and stability of the domains in solution. We monitored the membrane binding and penetration capacities of the domains, isolated or linked together as in the native toxin. We found that acidification stabilizes the domains. Unexpectedly LC interacts with and permeabilizes model membranes more efficiently than H_N_, H_N_ further enhances the stabilization of LC induced by acidic pH and acts as a moderator of LC in its interaction with the membrane.

## Methods

### Cloning, production, and purification of recombinant LC, H_N_ and tandem LC-H_N_ constructs of BoNT/A

Cloning of the Tm fragment, corresponding to the sequence encoding for fragment C454/S-S877 of H_N_, has been described previously [23]. A synthetic gene encoding residues M1-S877 of both LC and H_N_ domains of BoNT/A (named LC-H_N_) was purchased from Geneart (Regensburg, Germany) and cloned in a pCR-Script plasmid. The sequence was optimized for the expression in *Escherichia coli*. A potential Shine Delgarno sequence was also mutated. Two restriction sites for BamHI at the 5’ end and for AvaI at the 3’ end were introduced. The plasmid pCR-Script encoding for LC-H_N_ was amplified and then digested by BamHI and AvaI. The fragment corresponding to LC-H_N_ was cloned in the pQE-81L (Qiagen) plasmid digested also by BamHI and AvaI. LC was obtained by site directed mutagenesis on the pQE-81L-LC-H_N_ plasmid in order to introduce a stop codon at position 426 of LC-H_N_. The primers used for mutagenesis were 5’-CCGGCCTGTTCGAATTCTAaAAACTGCTGTGTGTTCGTGGC-3’ and 5’-GCCACGAACACACAGCAGTTTtTAGAATTCGAACAGGCCGG-3’. All plasmids, whose sequence was verified, code for a recombinant protein with a His-tag at the N-terminal end. The *E*. *coli* strain M15 was used as the host for recombinant protein production.

Expression and purification of the Tm fragment of H_N_ has been described previously [23]. The Tm protein corresponds to the activated form of the BoNT/A H_N_, i.e. H_N_ following proteolytic cleavage between LC and HC, before residue C454 (S1 Fig).

We have chosen to work with 1–425 LC since 1–448 LC was reported to precipitate upon storage [24]. 1–425 LC was shown to have proper structure and enzymatic activity [25,26]. LC of BoNT/A expression was performed by inoculation of 1 L of Terrific Broth medium (Difco, Detroit, USA) with 15 mL of an overnight culture performed at 28°C. Induction was started at an optical density at 600 nm (OD_600_) of 0.8 by addition of 1 mM of isopropyl-1-thio-b-D-galactopyranoside and carried out overnight at 16°C. The cultures were centrifuged at 5,000 g for 45 min and the pellet was solubilized in a 20 mM Tris/HCl, 500 mM NaCl, 10 mM imidazole buffer at pH 7.9. Recombinant proteins were extracted by biochemical lysis of the bacteria with 10 mg of lysozyme for 1 hour followed by mechanical lysis using a cell disruption apparatus (Constant Systems Ltd, UK). LC was purified from the soluble fraction of the lysate using a His-Trap HP 5 mL column and an Äkta purifier system (GE Healthcare, USA). The protein was eluted with a 20 mM Tris/HCl, 500 mM NaCl, 250 mM imidazole buffer at pH 7.9. The sample was then subjected to a dialysis in a 10 mM Tris/HCl, 20 mM NaCl buffer at pH 7.8 before cation exchange chromatography using a Hi-TrapQ HP 5 mL column (GE Healthcare, USA). The protein was eluted with a 10 mM Tris/HCl, 150 mM NaCl buffer at pH 7.8.

The expression of LC-H_N_ was performed by inoculation of 1 L of Terrific Broth medium with 25 mL of an overnight culture performed at 37°C. At an OD_600_ of 0.3, the culture was placed at 15°C. The induction was started at an OD_600_ of 0.6 by addition of 1 mM of isopropyl-1-thio-b-D-galactopyranoside and carried out overnight at 15°C. The culture was centrifuged at 5,000 g for 45 min and the pellet was solubilized in a 20 mM Na_2_HPO_4_/NaH_2_PO_4_, 500 mM NaCl, 10 mM imidazole buffer at pH 8. Recombinant proteins were extracted by biochemical lysis with 10 mg of lysozyme for 1 hour followed by mechanical lysis using a cell disruption apparatus (Constant Systems Ltd, UK). LC-H_N_ protein was purified from the soluble fraction of the lysate using a His-Trap HP 5mL column and an Äkta purifier system. The protein was eluted with a 20 mM Na_2_HPO_4_/NaH_2_PO_4_, 500 mM NaCl, 50 mM imidazole buffer at pH 8. The sample was then subjected to a size exclusion chromatography using a Sephacryl S200 26/10 300 mL column (GE Healthcare, USA) with a 20 mM sodium phosphate, 150 mM NaCl, buffer at pH8.

For all proteins, purification buffer was exchanged by dialysis with a 5 mM phosphate/citrate buffer at pH 7 and were kept at -20°C for LC-H_N_ and H_N_ and at -80°C for LC. Protein concentration was performed by measuring the absorbance of the protein at the ultraviolet wavelength of 278 nm and using Beer-lambert’s law. Molar extinction coefficient of each protein was determined from the protein sequence on the Protein Calculator web resource (http://protcalc.sourceforge.net).

Coomassie blue staining of the proteins following SDS-PAGE showed that they had been purified nearly to homogeneity. The catalytic activity of LC and the protein LC-H_N_ was assayed using a fluorogenic peptide mimic of the substrate of BoNT/A [25].

### Lipid vesicles

Lipids were purchased from Avanti Polar Lipids (Alabaster, USA). Suspensions of large unilamellar vesicles (LUV) were prepared in 5 mM phosphate/citrate buffer at pH 7 with egg phosphatidylcholine (EPC, 840051) and egg phosphatidic acid (EPA, 840101) at a 9/1 molar ratio for anionic vesicles. After evaporation of the chloroform used for their solubilization, lipids were suspended in phosphate/citrate buffer and then subjected to 5 cycles of freezing in liquid nitrogen followed by thawing at room temperature. This suspension was then filtered twice on a 0.4 μm filter and twice on a 0.2 μm filters using a homemade extruder. The size of the LUV was checked by dynamic light scattering at 830 nm by using DynaPro-TC-04 DLS equipment (Protein Solutions, Wyatt Technology, Santa Barbara, CA, USA) and found to be between 140 nm and 160 nm.

### Experimental buffers

Proteins were kept in 5 mM phosphate/citrate buffer at pH 7 and diluted in a series of phosphate/citrate buffers with varying pH. Permeabilization experiments were performed in 5 mM phosphate/citrate, 100 mM NaCl buffer of various pH. The concentration of protein was checked by measuring the absorbance at 278 nm. The pH of the diluted protein samples was checked before and after each experiment.

### Circular dichroïsm (CD) spectropolarimetry

CD experiments were performed on a CD spectropolarimeter model 215 (Aviv, USA). The scans were recorded at a scan rate of 1 nm/s (step, 1 nm; integration time, 1s) with a time constant of 100 ms and a bandwidth of 1 nm. Each spectrum was an average of 5 scans and 3 scans for near-UV and far-UV CD spectra, respectively. A rectangular quartz Suprasil cell of 10 mm path length and a cylindrical quartz Suprasil cell of 0.2 mm path length (104B-QS and 121.000-QS, Hellma, Germany) were used for recording CD activity in near-UV and far-UV regions, respectively. LC, H_N_ and LC-H_N_ were diluted to 1 mg/mL for each protein (corresponding to 10 μM for LC-H_N_ and 20 μM LC and H_N_). The protein concentration (absorbance at 280 nm, Beer-Lambert’s law) and pH were also checked after measurement. A 5 mM phosphate/citrate pH 5 dialysis buffer was used as blank in far-UV and near-UV regions and its spectrum was subtracted from protein CD spectra. Data were normalized to the molar peptide bond concentration and path length and expressed as Mean Residue Ellipticity per amino acid ([θ] degree·cm^2^·dmol^-1^.aa^-1^) for far-UV CD and as Mean Residue Ellipticity ([θ] degree.cm^2^.dmol^-1^) for near-UV CD.

### Small angle X-ray scattering (SAXS) experiments

SAXS samples were prepared in 5 mM phosphate/citrate, 150 mM NaCl pH 7 or pH 4. The initial protein concentration of the samples was 10 mg/ml. Prior to data collection, these samples were injected on the online HPLC system of the SWING Beamline at Synchrotron SOLEIL (Gif-sur-Yvette) to eliminate aggregates. Synchrotron radiation X-ray scattering data were collected at 17°C following standard procedures of the SWING Beamline and were processed with FOXTROT (from SWING beamline) and PRIMUS (Primary analysis and Manipulation with Small Angle Scattering DATA) from the ATSAS package (http://www.embl-hamburg.de/biosaxs/software.html). The scattering curves were corrected from scattering due to the buffer and Guinier plots corresponding to each curve were generated (S2 Fig). The pair-distribution functions were computed using the program GNOM from the ATSAS package, allowing determining the radius of gyration and the maximal distance of the molecule.

### Analytical ultracentrifugation (AUC)

The protein samples in 5 mM phosphate/citrate, 150 mM NaCl pH 7 or pH 4 were centrifuged at 36,000 rpm in a Beckman Coulter XL-I analytical ultracentrifuge at 20°C. Detection of the protein concentration as a function of radial position and time was performed by optical density measurements at a wavelength of 280 nm. Sedimentation velocity analysis was performed by continuous size distribution analysis c(s) with Sedfit 12.0 [27]. The following parameters were calculated using Sednterp 1.09 (http://www.jphilo.mailway.com/download.htm) and used to analyze experimental data: partial specific volume 0.738 mL.g^−1^, viscosity 0.01016 Poise and density 1.0044 g.mL^−1^. The sedimentation coefficients were corrected for viscosity and expressed as *s* (value at 20°C in water).

### Fluorescence spectroscopy

Tryptophan fluorescence measurements were performed with LC, H_N_ and LC-H_N_ in the absence or presence of LUV. Proteins were diluted to a final concentration of 1 μM in a 5 mM phosphate/citrate buffer with varying pH. Experiments with LUV were carried out using a protein/lipid molar ratio of 1/1000. After 2 hours of incubation, fluorescence measurements were performed with an FP-750 spectrofluorimeter (Jasco, Tokyo, Japan). Excitation wavelength was 295 nm. A bandwidth of 5 nm was used for both excitation and emission beams. The emission spectra were recorded from 310 to 370 nm at a scan rate of 125 nm/min. The fluorescence intensity ratio at 360 nm over 320 nm (rFI_360/320_) represents the average of three values obtained from emission spectra that were corrected for blank measurements [23,28,29].

### FRET experiments

LUV containing dansyl (*N*-(5-DimethylAminoNaphthalene-1-SulfonYL) were prepared from EPC, EPA, and dansyl-DHPE (*N*-(5-dimethylaminonaphthalene-1-sulfonyl)-1,2-DiHexadecanoyl-*sn*-glycero-3-PhosphoEthanolamine, D57, Molecular probes, Life technologies, USA) at a 9/1/0.5 molar ratio. LUV were then prepared as described above. A rectangular Quartz Suprasil cell of 10 mm path length was used for recording the fluorescence transfer between protein tryptophan donors and the dansyl acceptor. LC, H_N_ or LC-H_N_ were diluted to a final concentration of 0.5 μM and mixed with LUV at protein/lipid molar ratio of 1/100 in a 5 mM phosphate/citrate buffer at various pH. Control samples without protein were prepared. After 2 hours of incubation fluorescence measurements were carried out with an FP-750 spectrofluorimeter (Jasco, Tokyo, Japan). Excitation and emission wavelengths were 292 nm and 520 nm, respectively. Bandwidths of 5 nm were used for both excitation and emission beams. The emission spectra were recorded from 500 to 540 nm at a scan rate of 125 nm/min. Each emission value at 520 nm is the average calculated from 3 emissions scans. The fluorescence intensity of the dansyl at the plateau was normalized to its initial fluorescence in the absence of protein [23,28]. Results represent the average of four independent measurements for each protein.

### LUV permeabilization assay

LUV composed of EPC/EPA at 9/1 molar ratio were prepared as described above, in a 5 mM phosphate/citrate, 100 mM NaCl pH 7 buffer containing 50 mM sulforhodamine B (a self-quenching concentration). After extrusion, unincorporated dye was removed by size exclusion chromatography on a PD10 column (Amersham Biosciences, USA) equilibrated with 5 mM phosphate/citrate, 50 mM or 100 mM NaCl pH 7 buffer. Dye efflux was monitored on a Jasco FP-750 spectrofluorimeter by measuring the increase in fluorescence after adding of 0.1 μM of protein to a suspension of LUV at 100 μM lipids in 5 mM phosphate/citrate, 100 mM NaCl buffer at different pH with stirring. Excitation and emission wavelengths were 569 nm and 586 nm, respectively. Bandwidths of 5 nm were used for both excitation and emission beams [23].

Fluorescence was normalized with the following equation:

$${\text{F(t)}}_{\text{norm}}=(\text{F}(\text{t})-{\text{F}}_{0})/({\text{F}}_{\text{max}}-{\text{F}}_{0})$$ (1)

where F_0_ is the fluorescence level before protein addition and F_max_ the level after addition of Triton X-100 at the end of each assay [23] (S3 Fig).

### Chemical protein denaturation by guanidine hydrochloride (GdnHCl)

Stock solutions of 5 mM phosphate/citrate and 7 M GdnHCl were prepared, their pH were adjusted using HCl and NaOH, and filtered. The refractive index of each solution was measured to check the precise GdnHCl concentration (R-5000 hand refractometer (Atago, Japan)). A range of solutions with GdnHCl concentrations varying from 0 to 6.8 M for each studied pH were prepared by dilution of stock solutions in 5 mM phosphate/citrate at the same pH as the stock solution [30]. LC, H_N_ and LC-H_N_ proteins were diluted to a final concentration of 1 μM in 1.5 mL with the complete set of GdnHCl solutions. Samples were incubated overnight before fluorescence measurements on a Jasco FP-750 spectrofluorimeter. Excitation wavelength was 295 nm with a bandwidth of 5 nm for both excitation and emission beams. The emission spectra were recorded from 310 to 370 nm at a scan rate of 125 nm/min. Fluorescence intensity ratio at 360 nm over 320 nm (rFI_360/320_) were determined. These wavelengths are related to tryptophan fluorescence intensities in apolar (320 nm) and solvated (360 nm) environments, respectively, and the values of rFI_360/320_ are therefore very sensitive to weak polarity changes in the tryptophan surroundings. Each data point represents the average of three values obtained from emission spectra that were corrected for blank measurements. rFI was plotted as a function of GdnHCl concentration to obtain the GdnHCl-unfolding curves [31]. Curves were analyzed assuming a two-state mechanism and were fitted with the following equation:

$$\text{y(D)}=1/(1+\text{exp}((m\text{D}-\Delta {\text{G}}^{0})/\text{RT}))$$ (2)

where ΔG^0^ is the free energy in the absence of chaotropic agent, D the denaturant concentration and *m* the coefficient of dependence of free energy on the denaturant concentration. *m* is related to the variation of the solvent-accessible surface area between the folded and unfolded states. D_1/2_, the denaturant concentration required to achieve half denaturation was deduced from the expression of free energy at half denaturation:

$$\Delta \text{G}=-\text{RTlnK}=-\Delta {\text{G}}^{0}+m{\text{D}}_{1/2}=0$$ (3)

Hence,

$${\text{D}}_{1/2}=\Delta {\text{G}}^{0}\text{/}m$$ (4)

## Results

**Fig 1:**
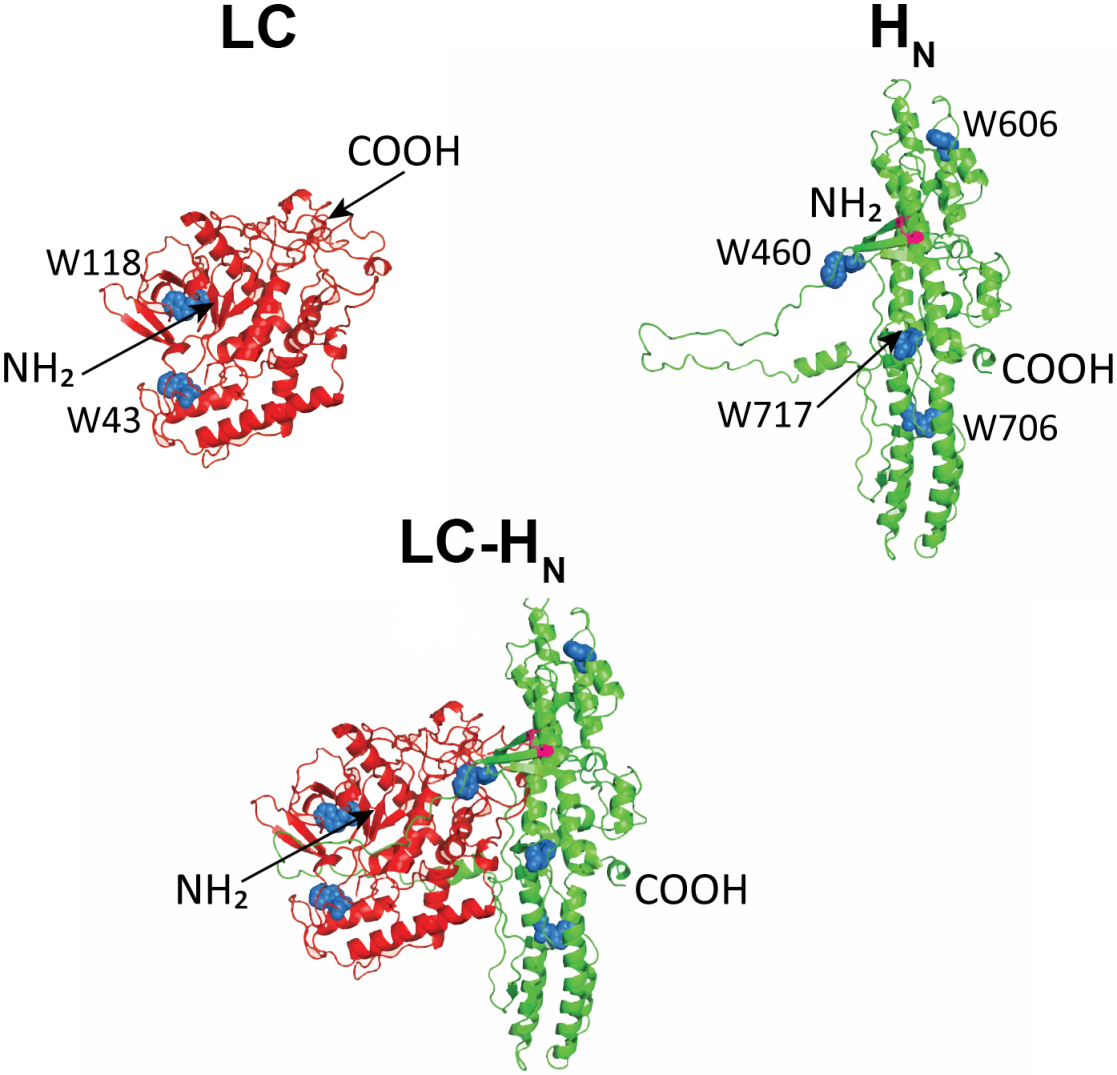
Structure of the recombinant LC, H_N_ and LC-H_N_ proteins of BoNT/A. Ribbon representations of LC, H_N_ and LC-H_N_ predicted from the structure of BoNT/A (Protein Data Bank file: 3BTA). The N- and C-terminal ends are indicated (NH_2_ and COOH) for each protein. The Trp residues are represented as blue spheres for the three proteins and indicated in C as W43, W118 and as W460, W606, W706 and W717 for H_N_.

Three protein constructs were used in this study: LC and H_N_, corresponding to the isolated catalytic and translocation domains of BoNT/A, and LC-H_N_, corresponding to a truncated BoNT/A toxin lacking the H_C_ domain (Fig 1). In the LC-H_N_ protein construct, LC and H_N_ are linked through a disulfide bond as in the native toxin. The peptide chain linking the domains is cleaved by incubation with trypsin. Cleavage of the peptide chain and integrity of the inter-domain disulfide bond were checked by SDS-PAGE under reducing and non-reducing conditions, and Coomassie blue staining (S1 Fig).

### LC and HN of BoNT/A do not undergo any major conformational change upon acidification in solution

**Fig 2:**
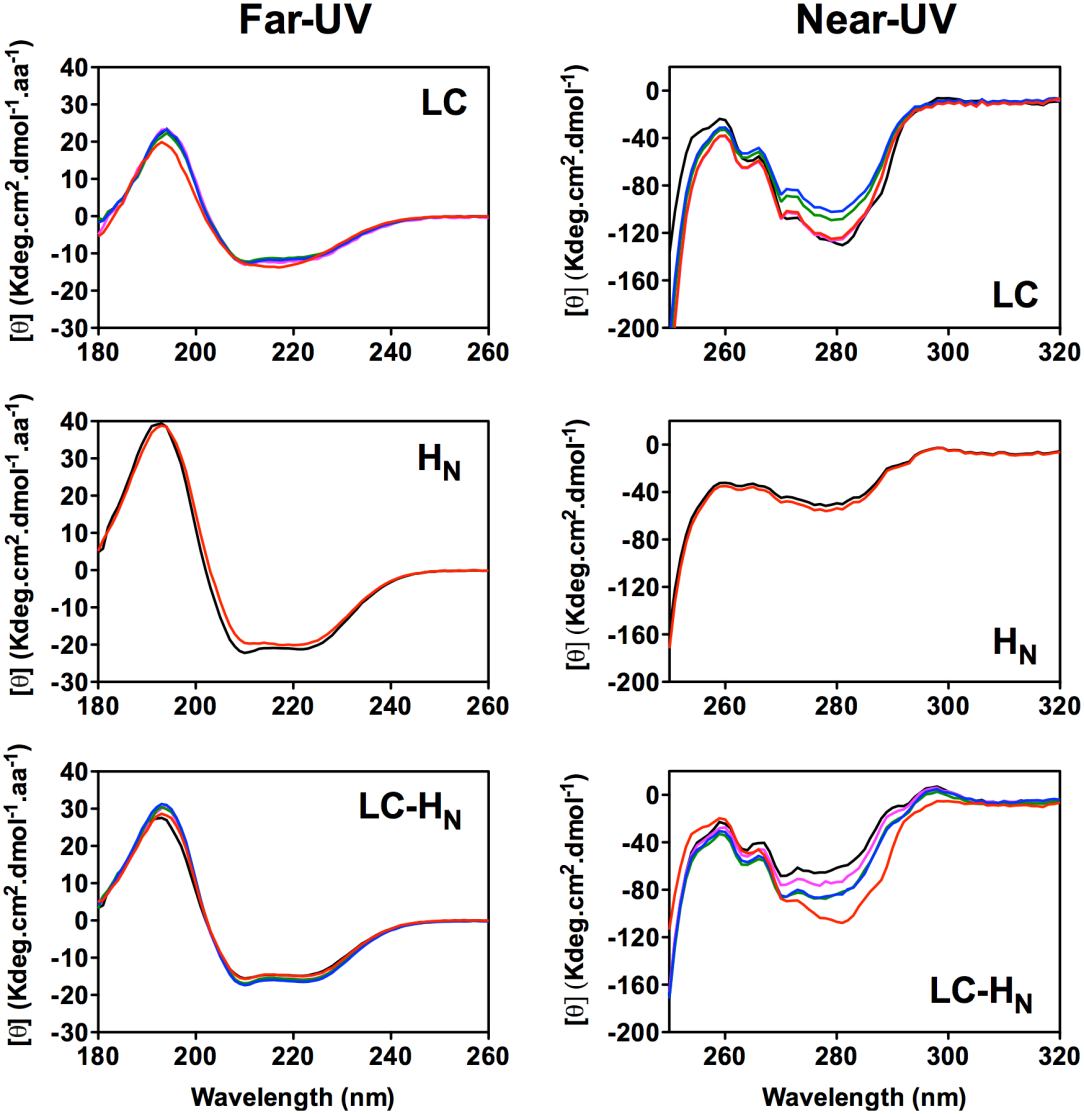
Far and near-UV CD spectra of LC, H_N_ and LC-H_N_ proteins of BoNT/A. Left, Far-UV and right Near-UV at various pH, pH 7 (black curve); pH 6 (pink curve); pH 5 (green curve); pH 4 (blue curve) and pH 3 (red curve).

The structure of the proteins constructs was investigated by circular dichroïsm (CD) in the far- and near-UV as a function of the pH. In the far-UV (180–260 nm) the CD signal measured for a protein is due mostly to the peptide bonds, so the spectra are indicative of its secondary structure. The far-UV spectra of the three proteins display π-π and n-π characteristics of a α-helical content (Fig 2). Analysis of the secondary structure of the three proteins from these CD data is in agreement with the crystal structure of BoNT/A (Fig 1 and Table 1) [4]. The protein CD signal in the near-UV (250–320 nm) arises from aromatic amino acid side chains constrained in a tertiary structure. LC spectra show a strong negative signal around 280 nm that may be attributed to Tyr or Trp side chains and two smaller signals at 262 nm and 268 nm that may be attributed to Phe side chains. The spectra of H_N_ display some weak signals in the absorption regions of Phe, Tyr and Trp side chains as observed previously [23]. LC-H_N_ near-UV spectra seem to combine signals from both LC and H_N_.

**Table 1:** Comparison of the secondary structure of the three proteins based on the far-UV CD spectra at pH 7 (BestSel) with the secondary structure observed in the crystal structure of BoNT/A (3BTA) (STRIDE PDB).

	BestSel				STRIDE PDB			
	LC	H_N_	LC-H_N_	LC + H_N_ isolated	LC	H_N_	LC-H_N_	LC + H_N_ isolated
alpha	28.3	51.8	39.6	40.05	30.2	50.2	40.7	40.2
beta	17.3	7	14.2	12.15	14	1.4	9.7	7.7
Others^1^	54.4	41.2	46.2	47.8	55.8	48.4	49.6	52.1

^1^Others: turns and random coils

Upon acidification no remarkable change could be observed in the far-UV spectra for the three proteins. However, a moderate increase of the negative peak intensity around 280 nm in the near-UV region can be seen at pH 3 for LC and LC-H_N_. This suggests an increase of the tertiary structure constraints surrounding one or several aromatic residues within the LC chain, as it is shared by both proteins.

Altogether, the data show that LC, H_N_ and LC-H_N_ are folded in the range of pH 3–7 with secondary and tertiary structures compatible with those found in the crystal structure of the toxin (pdb 3BTA). Acidification does not trigger any visible conformational change, except around pH 3 within the LC and LC-H_N_ proteins. The data suggest a moderate gain of tertiary structure within LC without any indication of partial unfolding.

**Fig 3:**
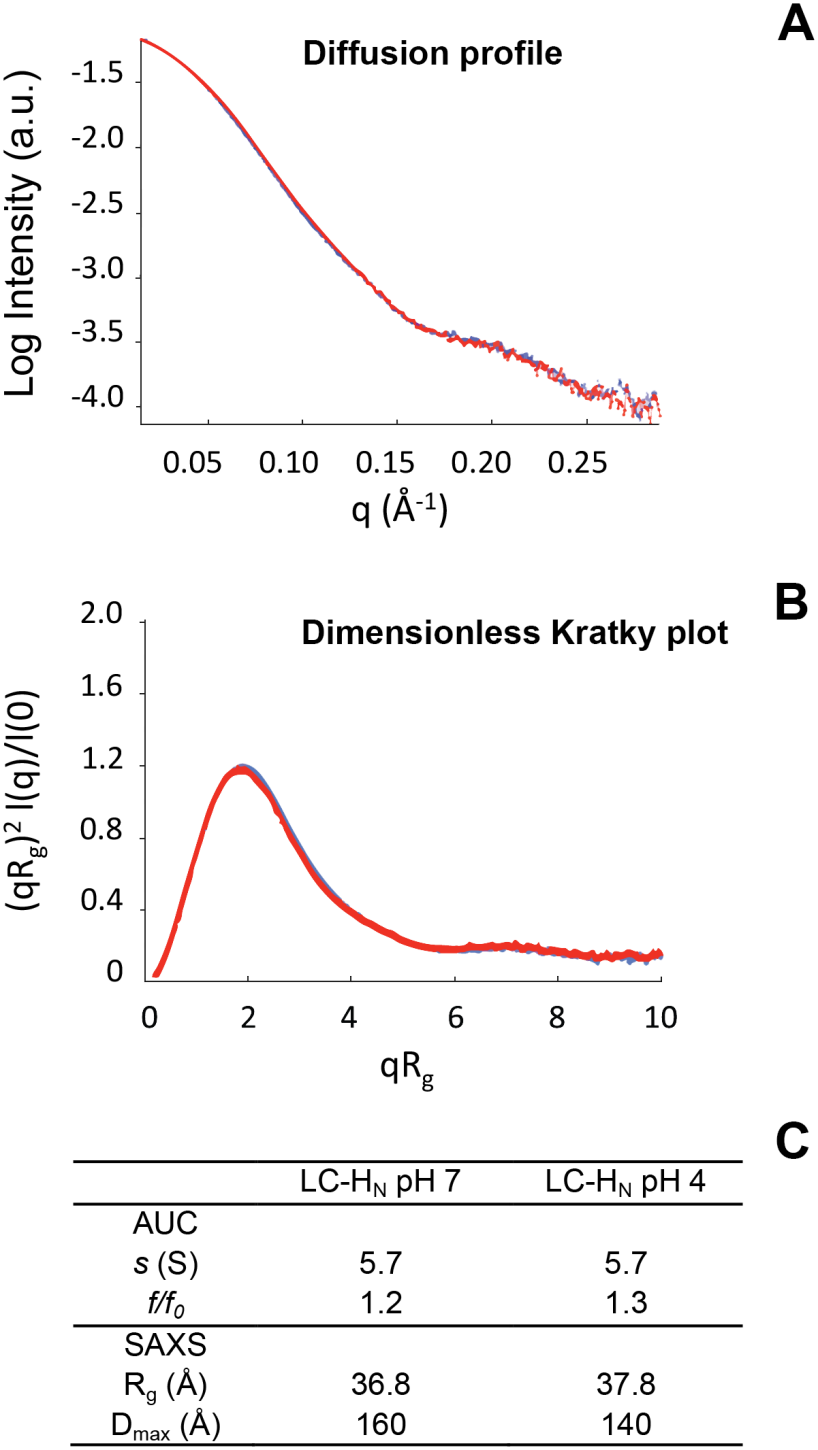
SAXS and analytical ultracentrifugation analysis of LC-H_N_ at pH 7 and pH 4. A, comparison of the superposition of the logarithm of the SAXS intensities as a function of the amplitude of the diffusion vector [f/f_0_ = 4πsinθ/λ], for LC-H_N_ at pH 7 (red curve) and pH 4 (blue curve). B, Superposition of the dimensionless Kratky plots of LC-H_N_ at pH 7 (red curve) and pH 4 (blue curve). This representation reveals the type of structure: compact, partially folded or unfolded. C, summary of shape describing parameters obtained from AUC and SAXS: sedimentation coefficient (*s*, in Svedberg), frictional ratio (*f/f*_*0*_), radius of gyration (R_g_).

Small Angle X-ray scattering (SAXS) and analytical ultracentrifugation (AUC) provide information on the shape of a protein and the formation of multimers (Fig 3). SAXS gives accurate information about the conformation in solution of macromolecules. The SAXS curves of LC-H_N_ at pH 7 and pH 4 are similar indicating that acidification does not trigger any change in the global fold of LC-H_N_ (Fig 3A). The normalized Kratky plots are characteristic of folded proteins (Fig 3B). The Inverse Fourier transform of the scattering intensity yields the distance distribution function, P(r). Two important parameters can be extracted from this curve: the radius of gyration (R_g_) and the maximum particle diameter (D_max_) of the protein R_g_ (Fig 3C). The R_g_ of LC-H_N_ at pH 7 and pH 4 are close, confirming that LC-H_N_ does not undergo important shape modification upon acidification. Consistently, AUC experiments showed similar sedimentation coefficients (*s*) for LC-H_N_ at neutral and acid pH (Fig 3C), suggesting that the overall shape of the protein is not modified by the acidification. In summary, SAXS and AUC data pointed out that the global shape of the LC-H_N_ protein remains the same and that no oligomerization occurred upon acidification.

### Acid pH stabilizes LC and HN of BoNT/A

**Fig 4:**
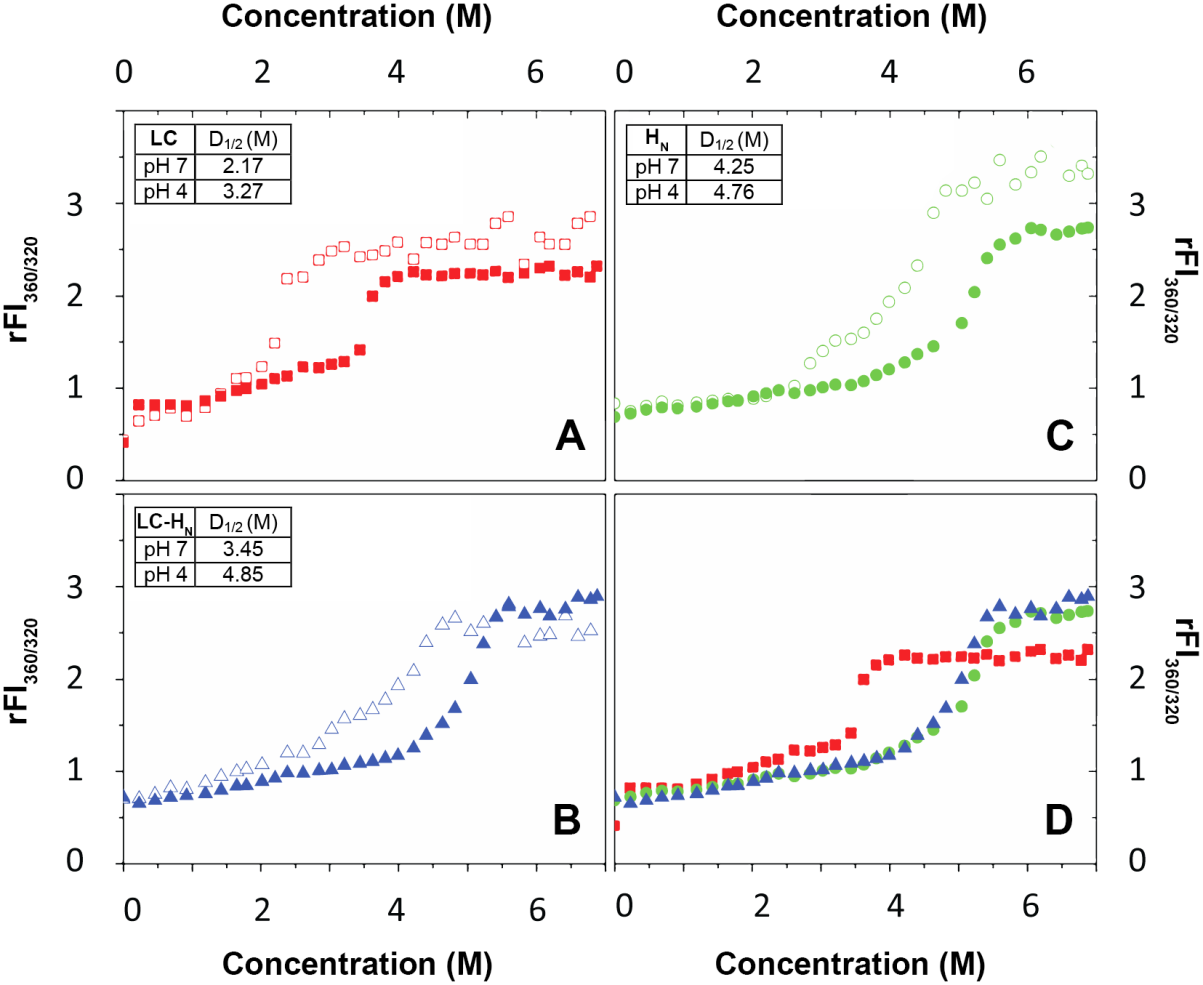
Effect of pH on the stability of LC, H_N_ and LC-H_N_ proteins. GdnHCl-induced denaturation of LC, H_N_ and LC-H_N_ followed by Trp fluorescence at pH 7 and pH 4. A, B, C, denaturation curves of LC, H_N_ and LC-H_N_ respectively, at pH 7 (open symbols) and pH 4 (closed symbols). D, superposition of the denaturation curves of LC (red squares), H_N_ (green circles) and LC-H_N_ (blue triangles) at pH 4. Inset, table of thermodynamic parameters ΔG^0^ and D_1/2_, for each protein at pH 7 and pH 4.

We investigated the stability of LC, H_N_ and LC-H_N_ as a function of pH by monitoring their unfolding in the presence of increasing concentrations of the chaotropic agent GdnHCl from 0 to 6.8 M (Fig 4). Chaotropic denaturation was preferred to thermal denaturation to avoid possible aggregation. Unfolding was monitored by measuring the ratio of the fluorescence signal at 360 nm and 320 nm of the Trp (rFI_360/320_ that reflects the polarity of Trp environment) as a function of pH. LC contains two Trp at positions 43 and 118 and H_N_ contains four Trp at position 460, 606, 707, and 717 (Fig 1). A rFI_360/320_ that shifts toward high values means that Trp become exposed to the solvent, due to the unfolding of the protein. All proteins exhibit a typical GdnHCl-induced denaturation from the folded state to the unfolded state at neutral pH (Fig 4). The denaturation curves show only a slight change in the fluorescence of the Trp between 0 and 2 M of GdnHCl. This indicates minor structural changes. A pH drop does not notably change the curves at these concentrations of denaturing agent. In contrast, at higher GdnHCl concentrations, a clear denaturation transition is observed for all three proteins (Fig 4 for pH 7 and pH 4). The rFI_360/320_ signal of LC-H_N_ is almost identical to the signal of H_N_ indicating that H_N_ is dominating the LC-H_N_ signal. This may be due to the fact that H_N_ contains four Trp as compared to LC that contains only two. In addition, H_N_ stabilizes LC (see below), further reducing the contribution of LC to the change of rFI_360/320_. Interestingly, the denaturation curves are shifted towards higher GdnHCl concentrations for all the proteins as pH drops (Fig 4 for pH 7 and pH 4). Thermodynamic parameters were extracted by applying a fitting procedure to the experimental data [31]. One or two folding intermediates may appear during denaturation as revealed by slight changes in the slope of the curves. However, fitting according to a three-state model could not be achieved and a two-state model was used as a best approximation. At neutral pH, 2.17 M, 4.25 M and 3.45 M of GdnHCl is needed to achieve half denaturation of LC, H_N_ and LC-H_N_, respectively (Fig 4, inset tables). These values of D_1/2_ increase as pH drops (shown Fig 4 for pH 4). At pH 4, these D_1/2_ concentrations are 3.27 M, 4.76 M and 4.85 M (Fig 4, inset tables), respectively. This is consistent with the increase of ΔG^0^ values extracted from the curves upon acidification. At pH 7 the ΔG^0^ are 3.7 kcal.mol^-1^, 3.3 kcal.mol^-1^ and 3.5 kcal.mol^-1^ for LC, H_N_ and LC-H_N_ respectively, whereas at pH 4 the values rise to 4.3 kcal.mol^-1^, 6.1 kcal.mol^-1^ and 5.7 kcal.mol^-1^, respectively. Interestingly, while the stability of LC slightly increases with the pH drop from 7 to 4 with a gain in ΔG^0^ of 0.6 kcal.mol^-1^, that of H_N_ is considerably increased with almost a doubling of its ΔG^0^. The situation for LC-H_N_ is close to that of H_N_ suggesting that the presence of H_N_ increases the stability of LC, especially at acidic pH. Altogether, the results show that LC is less stable than H_N_, and that acidic pH strongly increase the stability of H_N_, which then stabilizes LC within the LC-H_N_ protein.

### The isolated LC of BoNT/A interacts with a membrane without requiring the presence of H_N_

**Fig 5:**
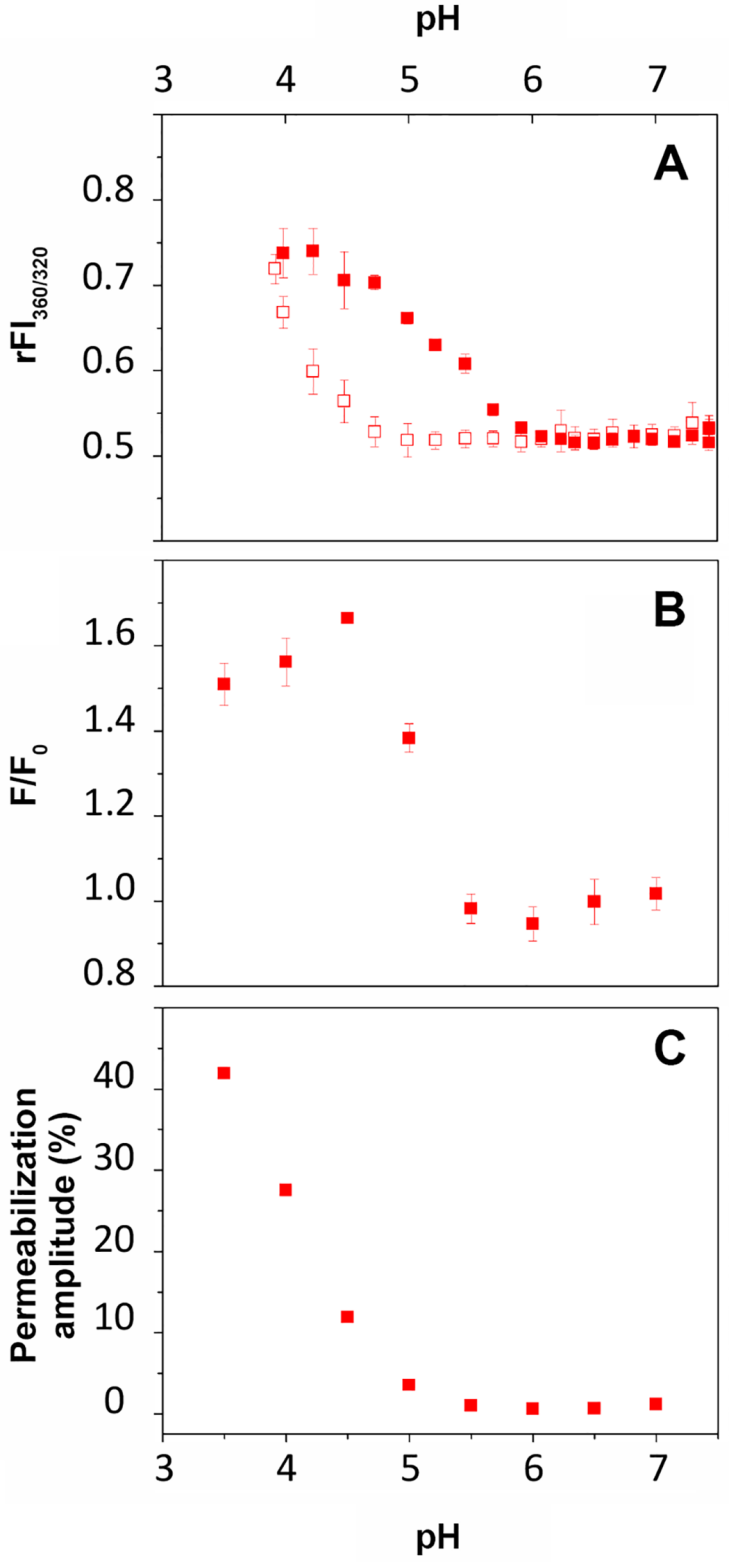
Membrane interaction of LC of BoNT/A as a function of pH. A, ratio of Trp fluorescence intensities at 360 nm and 320 nm in solution in absence (open squares) and in presence of LUV (closed squares). B, FRET from LC protein (0.5 μM) to dansyl-DHPE-containing LUV (50 μM). C, LUV (100 μM) permeabilization induced by LC (0.1 μM), expressed as percent of permeabilization amplitude reflected the maximum rate of permeabilization achieved for each pH.

We characterized previously the interaction of H_N_ of BoNT/A with negatively charged lipid vesicles [23]. Here, we investigated the capacity of the isolated LC to interact with a negatively charged membrane (LUV) in the absence of H_N_, as a function of pH. The interaction was monitored by three fluorescence methods: intrinsic fluorescence of the Trp of LC, FRET from the Trp of LC towards dansyl groups linked to the phospholipid head-groups of LUV and fluorescence dye recovery upon LUV permeabilization (Fig 5).

In the presence of LUV, the rFI_360/320_ increases as pH drops from 6 to 4.2 (Fig 5, top). In the absence of LUV, a similar transition occurs but at more than one pH unit lower. These results indicate that the environment of the Trp of LC in solution become more polar as pH decreases. The presence of membrane shifts the transition to higher pH values due to the partition of the protein from solution to the membrane.

When the Trp of the protein (donors) are close to the dansyl groups in the membrane (acceptor) FRET is detected as an increase of acceptor fluorescence. In the presence of LUV, the Trp intrinsic fluorescence transition we detected correlates nicely with an increase of the FRET signal from pH 6 to 4.5. The increase of the FRET signal upon acidification reveals a closer proximity between the Trp and the polar headgroups of the lipid bilayer and demonstrates the interaction of the protein with the membrane. Together, these data indicate that LC interacts progressively with the LUV as pH drops from 6 to about 4.

**Fig 6:**
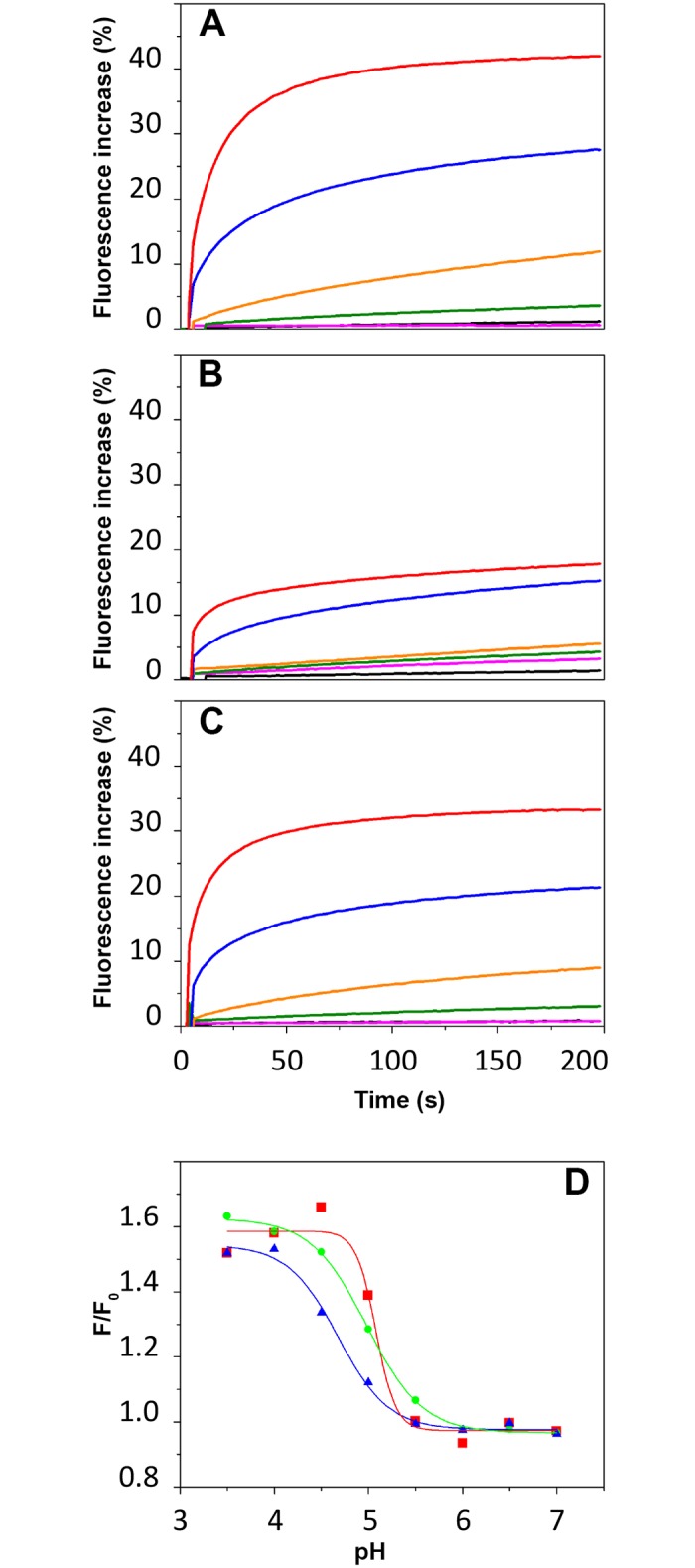
Comparison of permeabilization of, and membrane binding to LUV by LC, H_N_ and LC-H_N_ proteins of BoNT/A. A, B, C, Kinetics of SRB leakage from LUV (100 μM) after addition of LC (A), H_N_ (B) and LC-H_N_ (C) (0.1 μM) at pH 7 (black), pH 6 (pink), pH 5 (green), pH 4.5 (orange), pH 4 (blue) and pH 3.5 (red). S3 Fig shows absence of SRB leakage in the absence of proteins and 100% SRB release after Triton X-100 addition. D, FRET from LC (red), H_N_ (green) and LC-H_N_ (blue) at 0.5 μM to dansyl-DHPE-containing LUV at 50 μM.

LC exhibits a pH-dependent capacity to permeabilize the LUV as shown by its ability to induce the release of sulforhodamine B entrapped at self-quenching concentration in the LUV. The efficiency of permeabilization is expressed as the maximum percentage of permeabilization induced by LC at a given pH (Fig 5, bottom and Fig 6, top). The permeabilization phenomenon starts below pH 5.5, 0.5 pH units lower than the appearance of the FRET signal. Altogether, the data suggest that the interaction of LC with the lipid bilayer involves at least two steps: partition of LC into the interfacial region of the membrane (FRET signal without permeabilization) followed by a deeper penetration into the bilayer, which induce leakage of the dye contained in the LUV (permeabilization).

No permeabilization was observed with negative LUV in the presence of increased NaCl concentrations (200 mM instead of 100 mM). Similarly, no permeabilization was observed with LUV made of neutral phospholipids only (S4 Fig). This indicates that permeabilization involves electrostatic interactions between the proteins and negative charges on the membrane.

It is noteworthy that this partition-permeabilization process can occur in the absence of H_N_.

### H_N_ of BoNT/A moderates the propensity of LC to interact with membranes

Permeabilization and FRET experiments were also performed with the isolated H_N_ and the protein LC-H_N_ in order to compare the behavior of both domains isolated or linked by the native inter-domain disulfide bond. Fig 6A–6C show the kinetics of dye release from LUV in the presence of LC, H_N_ and LC-H_N_ at various pHs from 7 to 3.5. The results are expressed as the percentage of fluorescence increase after addition of the protein to the LUV, with 100% corresponding to the maximum release obtained by triton X-100. The three proteins show a capacity to destabilize the membrane as pH drops. H_N_ starts to permeabilize the LUV below pH 5, while the phenomenon starts below pH 5.5 for LC and LC-H_N_. However, LC was about twice more efficient than H_N_ in permeabilizing the LUV. While maximum dye release reached 40% for LC, it reached about 15% for H_N_ and 30% for LC-H_N_. In addition, the transition observed by FRET for the interaction with the membrane (Fig 6D) is more cooperative for LC than for H_N_, an intermediate situation being found for LC-H_N_.

Altogether, the data suggest that H_N_ moderates the propensity of LC to interact with and permeabilizes membranes at acidic pH.

## Discussion

How does BoNT/A, a soluble protein, manage to interact with and further penetrate into a membrane is a key issue for its translocation. Here we designed a study using a combination of spectroscopic methods to probe the effect of the pH on the structural properties and membrane interaction of the LC and H_N_ of BoNT/A, alone or covalently linked together.

The acidic state in solution of LC and H_N_, competent for membrane interaction, is characterized by increased stability without any major conformational change. Upon acidification, no significant secondary or tertiary structure change in solution can be observed for either LC or H_N_, alone or covalently linked by the native inter-domain disulfide bond (Fig 2). This observation is in agreement with previous studies that reported no major structural change upon acidification for LC [32,33], H_N_ [23,34] or the entire toxin [35,36]. Here, in contrast with previous studies on the LC and the whole toxin, we investigated a wide range of pHs from 7 down to 3. Interestingly, a gain of tertiary constraints seems to occur for the LC-H_N_ protein at low pH (Fig 2). This observation is in agreement with the increase of stability found for this protein as pH drops (Fig 4). Overall, the data suggest that LC and H_N_, isolated or linked together, do not undergo large pH-induced conformational changes. Nevertheless, our observations do not exclude local conformational changes, which would favor membrane partitioning. Indeed, Trp fluorescence changes can be seen as pH drops both for LC (Fig 5, top panel, open squares) and H_N_ [23,34].

However, an important finding of the thermodynamic analysis is that the stability of the domains in solution is increased as pH drops. Indeed, GdnHCl-induced unfolding experiments showed that H_N_ is more stable than LC and that the stability of H_N_ is considerably increased at low pH, thus apparently contributing to an increased stability of LC within LC-H_N_.

LC in its acidic state interacts and permeabilizes anionic phospholipid membranes in the absence of H_N_. Tables 2 and 3 summarize the features of the acidic states of LC and H_N_ in solution and in the presence of membranes. Considering LC alone, the data strongly suggest that the presence of the membrane displaces the equilibrium toward the acidic state of LC and its transition into a membrane-bound state (Fig 5A and 5B). Then, further acidification provokes permeabilization of the membrane by LC, indicative of a state in which LC destabilizes the lipid bilayer (Fig 5, bottom and Fig 6, top). The striking feature of this interaction is that membrane permeabilization by LC occurs without the help of another domain or component of the toxin or cellular machinery [37,38]. This finding infers the following question: what is the role of H_N_ for membrane interaction and thereby for translocation?

**Table 2:** Features of LC and H_N_ upon acidification in solution.

Features in solution	LC	H_N_
Trp^1^	Exposed from pH 4.7 to 3.8	Buried from pH 6.2 to 3.8^2^
Secondary structure^3^	Unchanged	Unchanged
Tertiary structure^3^	Increased	Unchanged
Stability^4^	Increased	Increased
Influence over the other domain	?	Stabilization

^1^Trp fluorescence

^2^not shown

^3^CD

^4^GdnHCl denaturation

**Table 3:** Features of LC and H_N_ upon acidification in the presence of membranes (LUV).

Features in presence of LUV	LC	H_N_
Trp^1^	Exposed from pH 5.9 to 4.2	Buried from pH 6.2 to 3.8^2^
Membrane binding^3^	From pH 5.5 to 4.5	From pH 5.5 to 3.5
Membrane permeabilization^4^	From 5 to 3.5	From 4.5 to 3.5
Influence over the other domain	?	Moderates membrane interaction

^1^Trp fluorescence

^2^Not shown

^3^FRET

^4^Fluorescent dye release from LUV

H_N_ of BoNT/A moderates the propensity of LC to interact with membranes at acidic pH. The data obtained here with the LC-H_N_ protein bring three observations: H_N_ contributes to the stabilization of LC at neutral and acidic pH in solution, it reduces the cooperativity of the binding of LC with membranes and it reduces the efficiency of membrane permeabilization of the membrane by LC. It is likely that at a given pH the lipid bilayer destabilization by LC is reduced by the presence of H_N_. We found previously that this capacity of H_N_ was due to its N-terminal belt that surrounds LC blocking its catalytic site [23]. One could hypothesize that the stabilization of LC by H_N_ that we observed in solution, also occurs during interaction with the membrane, thereby limiting membrane penetration and destabilization by LC. Thus, H_N_ would act as a chaperone-like domain, probably both through its belt and the wide interfacial region between the two domains. These two contact surfaces between the domains certainly represent major constraints over LC.

LC and H_N_ of BoNT/A possess structural and physico-chemical properties distinct from that of other translocating toxins. This is inferred by a comparison with the mechanisms of action of other translocating toxins: monomeric (AB_RTC_) and multimeric (anthrax, binary and AB_5_ toxins). The enzymatic components of anthrax toxin need the help of the membrane protective antigen hepta/octamer that catalyzes the unfolding of these enzymatic components and offers them a protein pore for their passage through the membrane [39]. Ricin and AB_5_ toxins such as Shiga toxin and Cholera toxin hijack the ERAD system in the endoplasmic reticulum to translocate into the cytosol [37,38].

It has been shown by electron and atomic force microscopies that BoNT/B and E [40,41] form multimers. In our observations by SAXS and AUC, no multimers were found in solution whatever the pH. Multimerization may depend highly on experimental conditions. In any case, one cannot rule out yet whether multimers of BoNT must form to ensure translocation.

The catalytic domain (C) of the diphtheria toxin, which is also a RTC toxin, interacts and permeabilizes the membrane of LUV with much better efficacy with the help of the translocation domain (T) [19,42]. In this case, T acts as a chaperone stabilizing successively several partially folded states of the C domain. Further description of this process for BoNT/A would require the determination of the structure of translocation intermediates.

Both the C and T domains of diphtheria toxin, which correspond functionally to LC and H_N_, undergo remarkable conformational changes upon acidification (in solution and in presence of membrane) to achieve a molten globule state needed for the membrane interaction [43,44]. We show by CD and SAXS (Figs 2 and 3) that LC and H_N_ of BoNT/A do not display any major conformational changes upon acidification in solution. This observation is supported by other works [32,33,35,36]. In addition, we found that LC has by itself a high propensity to bind (Fig 5B) and penetrate (Fig 5C) a membrane at acidic pH. The results suggest that the role of H_N_ is to negatively regulate this propensity. We showed previously that the belt region of H_N_ is responsible for the negative regulation of the interaction of H_N_ with the membrane [23]. As a consequence, it is very likely that LC of BoNT/A interacts with both the membrane and H_N_ during the first steps of its translocation. This is compatible with the cleft model proposed earlier by Montecucco [15,22], who showed also that LC is able to bind phospholipids. Single channel conductance measurements showed that LC blocks the ion channel formed in the membrane by H_N_ for BoNT/A and BoNT/E during translocation [45]. Reconciliation of this model with our observations would imply that the channel made by H_N_ would be closed by the passage of LC between the lipids of the bilayer and one of the faces of H_N_ embedded in the membrane.

## Supporting Information

S1 Fig Coomassie blue stained SDS-PAGE of recombinant proteins used in this study. **A,** LC-H_N_, H_N_ and LC in reducing and non-reducing conditions (+ or—β-mercaptoethanol, (β-ME)). **B**; LC-H_N_ was treated with trypsin before mixing with loading buffer + or—β-ME and analyzed on the SDS-PAGE. On each gel, Lane 1 is a ladder of protein molecular weight markers shown in kDa. The results show that the proteolytic cleavage site between LC and H_N_ is uncleaved in the recombinant protein before trypsin treatment and cleaved after. The disulfide bridge linking both domains is intact as well and reduced by β-ME.
(TIF)

S2 Fig Guinier representations of the SAXS curves obtained for LC-H_N_ at pH 7 and pH 4. These representations were calculated using Primus. They correspond to the logarithm of the SAXS intensity (log I) as a function of the square of the diffusion vector amplitude (q^2^), plotted up to a q*Rg limit of 1.3. The slope of these representations represents the radius of gyration (Rg) of the molecule.
(TIF)

S3 Fig Representative control for the LUV permeabilization experiments. Self fluorescence quenching of SRB entrapped inside the LUV define 0% fluorescence. The LUV are incubated at varying pH for over 150 s. The absence of fluorescence increase indicates the absence of SRB leakage from the LUV (in the absence of protein addition). After 150 s, the addition of Triton X-100 solubilizes the membrane of all the LUV and releases the entrapped SRB, leading to dequenching and maximum fluorescence (100%).
(TIF)

S4 Fig Effect of LUV surface charge on membrane permeabilization by LC, H_N_ and LC-H_N_. Kinetics of LUV leakage of LC, H_N_ and LC-H_N_ at pH 7 (black lines), pH 5 (green), and pH 4.5 (orange) in presence of neutral LUV (left) or negatively charged LUV with 200 mM NaCl (right). The arrows indicate the addition of protein and triton X-100 that gives the maximum of dye release.
(TIF)

S1 Table Thermodynamic parameters for the GdnHCl-induced unfolding transitions of LC (A), H_N_ (B) and LC-H_N_ (C) protein of BoNT/A at pH 7, 6, 5, 4, 3.5. ΔG_0_ is the free energy in the absence of chaotropic agent, D is the denaturant concentration and *m* is the coefficient of dependence of free energy on denaturant concentration. *m* is related to the variation of the solvent-accessible surface area between the folded and unfolded states.
(DOCX)
