# Correction: The Translocation Domain of Botulinum Neurotoxin A Moderates the Propensity of the Catalytic Domain to Interact with Membranes at Acidic pH

**DOI:** 10.1371/journal.pone.0161743

**Published:** 2016-08-18

**Authors:** Anne Araye, Amélie Goudet, Julien Barbier, Sylvain Pichard, Bruno Baron, Patrick England, Javier Pérez, Sophie Zinn-Justin, Alexandre Chenal, Daniel Gillet

A reference was incorrectly omitted in Table 1. The authors have provided the reference and a corrected table here.

**Table 1:** Comparison of the secondary structure of the three proteins based on the far-UV CD spectra at pH 7 (BeStSel [46]) with the secondary structure observed in the crystal structure of BoNT/A (3BTA) (STRIDE PDB).

	BeStSel				STRIDE PDB			
	LC	H_N_	LC-H_N_	LC + H_N_ isolated	LC	H_N_	LC-H_N_	LC + H_N_ isolated
**alpha**	**28.3**	**51.8**	**39.6**	**40.05**	**30.2**	**50.2**	**40.7**	**40.2**
**beta**	**17.3**	**7**	**14.2**	**12.15**	**14**	**1.4**	**9.7**	**7.7**
**Others**^1^	**54.4**	**41.2**	**46.2**	**47.8**	**55.8**	**48.4**	**49.6**	**52.1**

^1^Others: turns and random coils

46. Micsonai A, Wien F, Kernya L, Lee YH, Goto Y, Réfrégiers M, Kardos J. Accurate secondary structure prediction and fold recognition for circular dichroism spectroscopy. Proc Natl Acad Sci U S A. 2015; 112: E3095–E3103. doi 10.1073/pnas.1500851112. PMID:26038575.
