# Supplementary figures and images for: The Translocation Domain of Botulinum Neurotoxin A Moderates the Propensity of the Catalytic Domain to Interact with Membranes at Acidic pH
Source: PLoS One. 2016 Apr 12;11(4):e0153401. doi: 10.1371/journal.pone.0153401 (PMC4829238; doi:10.1371/journal.pone.0153401)

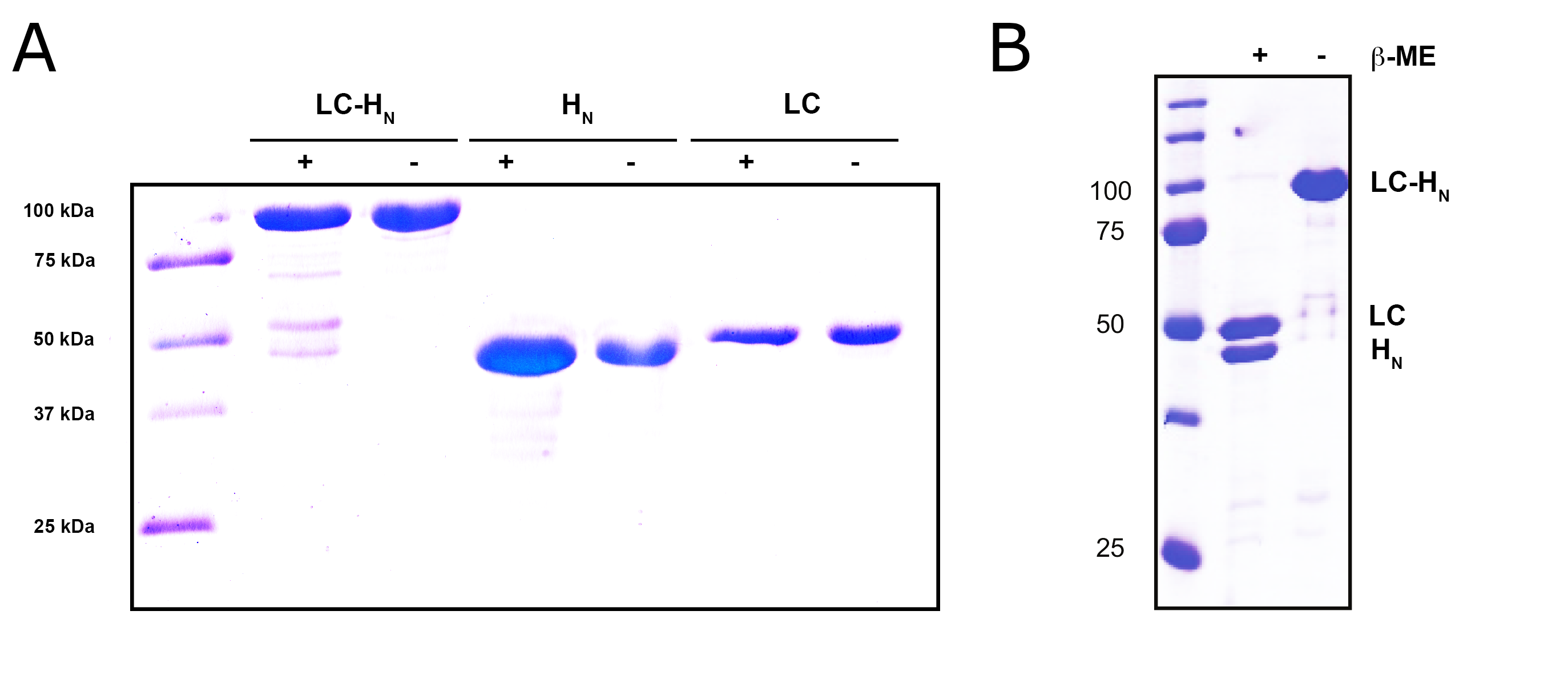

Supplement: S1 Fig — A, LC-HN, HN and LC in reducing and non-reducing conditions (+ or—β-mercaptoethanol, (β-ME)). B; LC-HN was treated with trypsin before mixing with loading buffer + or—β-ME and analyzed on the SDS-PAGE. On each gel, Lane 1 is a ladder of protein molecular weight markers shown in kDa. The results show that the proteolytic cleavage site between LC and HN is uncleaved in the recombinant protein before trypsin treatment and cleaved after. The disulfide bridge linking both domains is intact as well and reduced by β-ME. (TIF) [file pone.0153401.s001.tif]

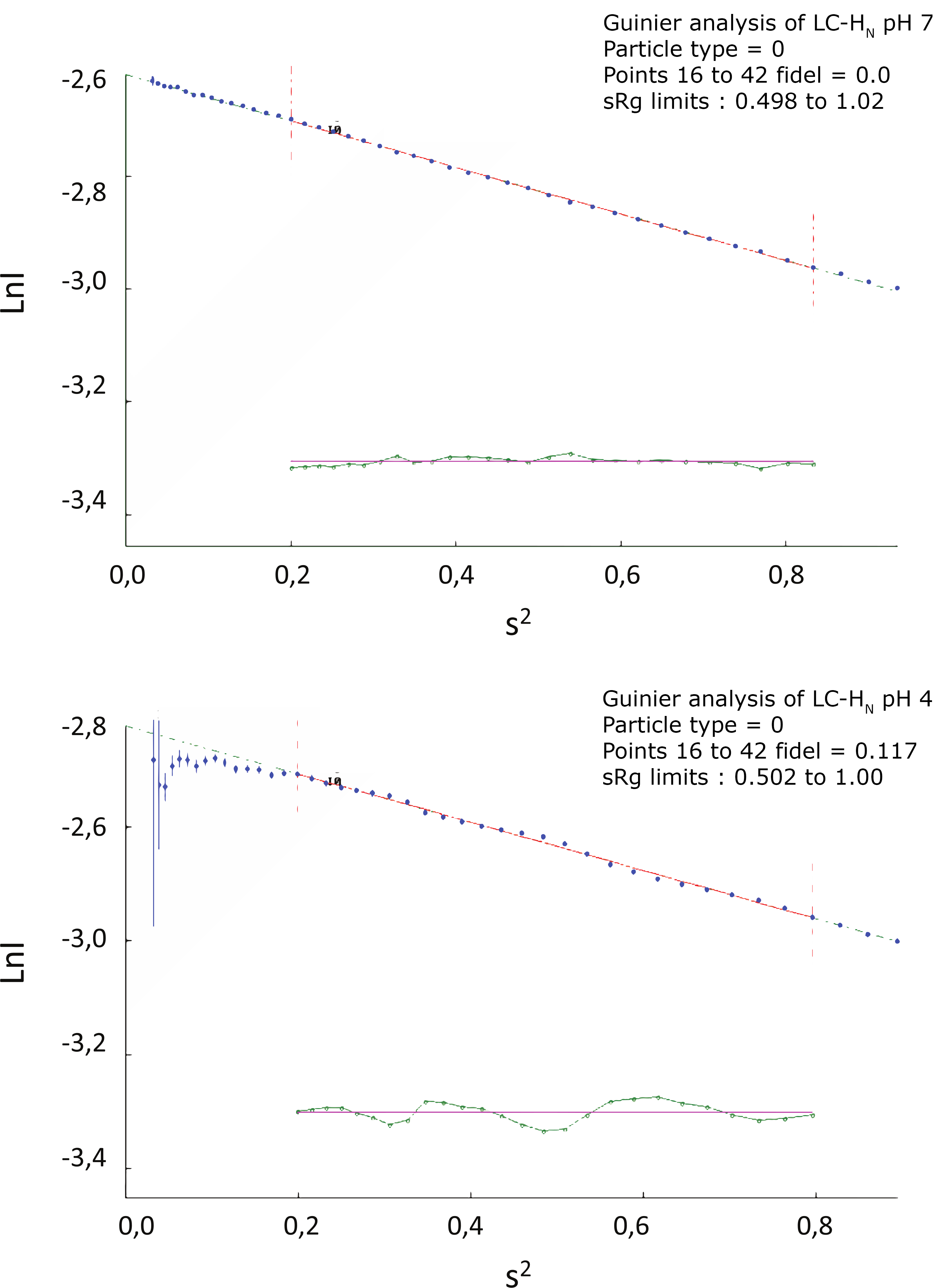

Supplement: S2 Fig — These representations were calculated using Primus. They correspond to the logarithm of the SAXS intensity (log I) as a function of the square of the diffusion vector amplitude (q2), plotted up to a q*Rg limit of 1.3. The slope of these representations represents the radius of gyration (Rg) of the molecule. (TIF) [file pone.0153401.s002.tif]

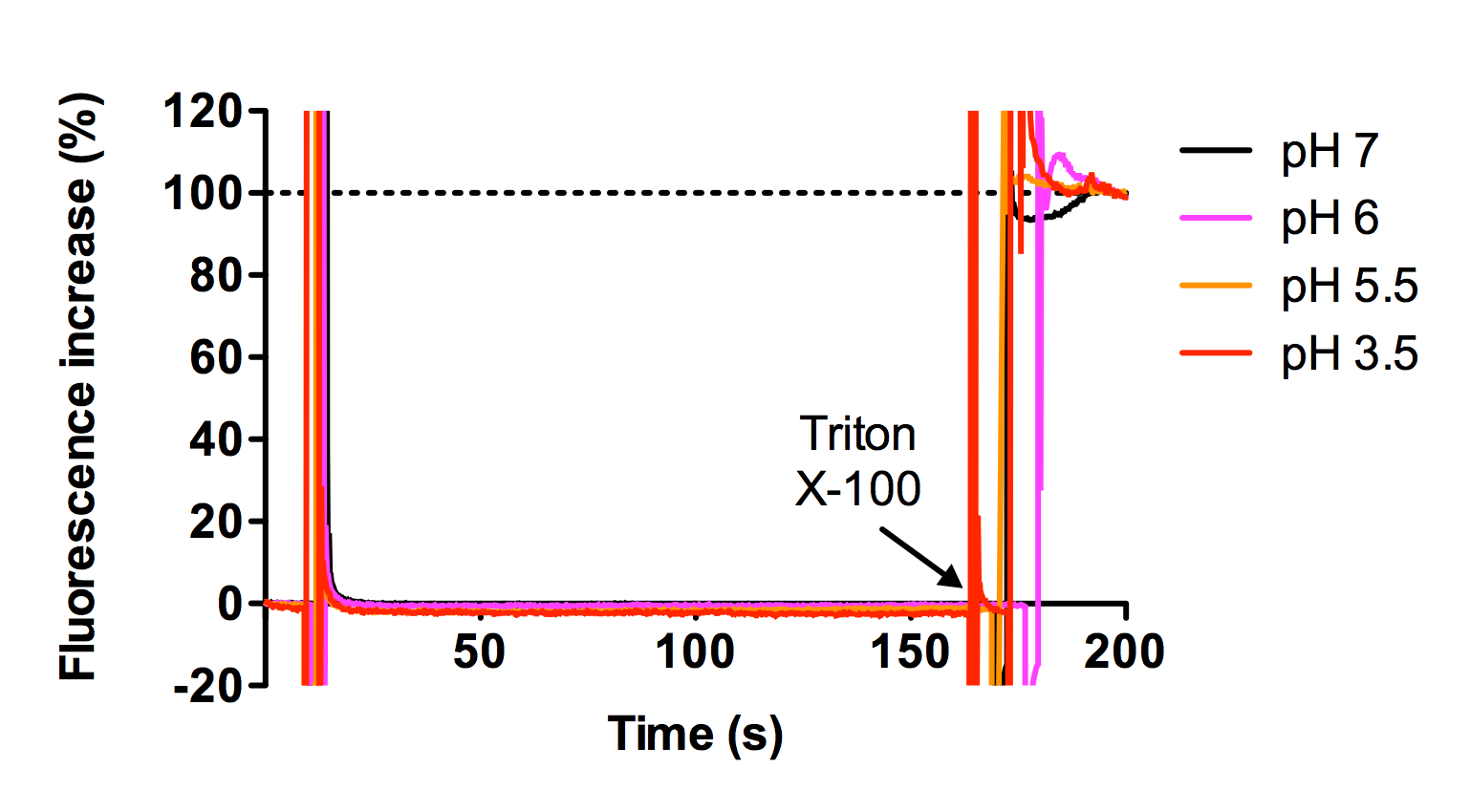

Supplement: S3 Fig — Self fluorescence quenching of SRB entrapped inside the LUV define 0% fluorescence. The LUV are incubated at varying pH for over 150 s. The absence of fluorescence increase indicates the absence of SRB leakage from the LUV (in the absence of protein addition). After 150 s, the addition of Triton X-100 solubilizes the membrane of all the LUV and releases the entrapped SRB, leading to dequenching and maximum fluorescence (100%). (TIF) [file pone.0153401.s003.tif]

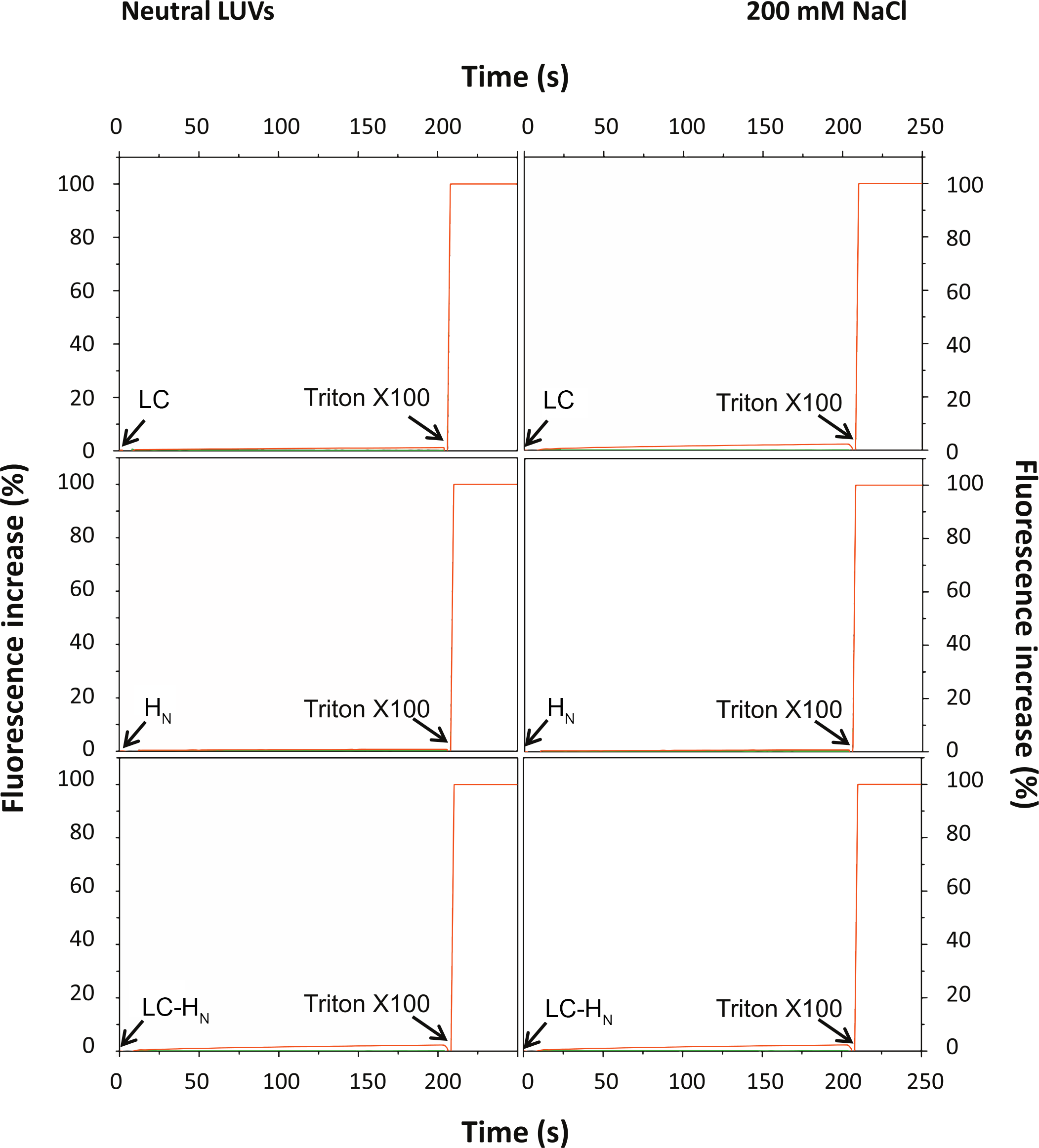

Supplement: S4 Fig — Kinetics of LUV leakage of LC, HN and LC-HN at pH 7 (black lines), pH 5 (green), and pH 4.5 (orange) in presence of neutral LUV (left) or negatively charged LUV with 200 mM NaCl (right). The arrows indicate the addition of protein and triton X-100 that gives the maximum of dye release. (TIF) [file pone.0153401.s004.tif]
