# Supplementary material for: The Translocation Domain of Botulinum Neurotoxin A Moderates the Propensity of the Catalytic Domain to Interact with Membranes at Acidic pH
Source: PLoS One. 2016 Apr 12;11(4):e0153401. doi: 10.1371/journal.pone.0153401 (PMC4829238; doi:10.1371/journal.pone.0153401)
Supplement: S1 Table — ΔG0 is the free energy in the absence of chaotropic agent, D is the denaturant concentration and m is the coefficient of dependence of free energy on denaturant concentration. m is related to the variation of the solvent-accessible surface area between the folded and unfolded states. (DOCX) [file pone.0153401.s005.docx]

| LC | ΔG^0^ (kcal.mol^-1^) | m (kcal.mol^-1^.M^-1^) | D_1/2_ (M) |
| --- | --- | --- | --- |
| pH 7 | 3.8 ± 0.8 | 1.7 ± 0.3 | 2.2 |
| pH 6 | 3.4 ± 0.4 | 1.5 ± 0.2 | 2.3 |
| pH 5 | 3.6 ± 0.6 | 1.3 ± 0.2 | 2.7 |
| pH 4 | 4.3 ± 0. 9 | 1.3 ± 0.3 | 3.3 |
| pH 3.5 | 5.3 ± 1.0 | 1.5 ± 0.3 | 3.6 |
|  |  |  |  |
| H_N_ | ΔG^0^ (kcal.mol^-1^) | m (kcal.mol^-1^.M^-1^) | D_1/2_ (M) |
| pH 7 | 3.9 ± 0.4 | 1.0 ± 0.1 | 4.1 |
| pH 6 | 3.8 ± 0.3 | 0.9 ± 0.1 | 4.1 |
| pH 5 | 5.3 ± 1.2 | 1.3 ± 0.3 | 4.2 |
| pH 4 | 5.0 ± 1.0 | 1.0 ± 0.2 | 4.8 |
| pH 3.5 | 5.0 ± 0.5 | 0.9 ± 0.1 | 5.4 |
|  |  |  |  |
| LC-H_N_ | ΔG^0^ (kcal.mol^-1^) | m (kcal.mol^-1^.M^-1^) | D_1/2_ (M) |
| pH 7 | 2.5 ± 0.4 | 0.7 ± 0.1 | 3.7 |
| pH 6 | 3.1 ± 0.4 | 0.9 ± 0.1 | 3.6 |
| pH 5 | 3.6 ± 0.3 | 0.9 ± 0.1 | 4.2 |
| pH 4 | 5.7 ± 0.8 | 1.2 ± 0.2 | 4.8 |
| pH 3.5 | 7.4 ± 1.8 | 1.4 ± 0.3 | 5.3 |
